# A Systems Biology Approach Identifies B Cell Maturation Antigen (BCMA) as a Biomarker Reflecting Oral Vaccine Induced IgA Antibody Responses in Humans

**DOI:** 10.3389/fimmu.2021.647873

**Published:** 2021-03-22

**Authors:** Lynda Mottram, Anna Lundgren, Ann-Mari Svennerholm, Susannah Leach

**Affiliations:** ^1^Gothenburg University Vaccine Research Institute (GUVAX), Department of Microbiology and Immunology, Institute of Biomedicine, Sahlgrenska Academy, University of Gothenburg, Gothenburg, Sweden; ^2^Department of Clinical Pharmacology, Sahlgrenska University Hospital, Gothenburg, Sweden

**Keywords:** global health, BCMA (TNFRSF17), enteric diseases, oral vaccines and futures, IgA responses, biomarkers

## Abstract

Vaccines against enteric diseases could improve global health. Despite this, only a few oral vaccines are currently available for human use. One way to facilitate such vaccine development could be to identify a practical and relatively low cost biomarker assay to assess oral vaccine induced primary and memory IgA immune responses in humans. Such an IgA biomarker assay could complement antigen-specific immune response measurements, enabling more oral vaccine candidates to be tested, whilst also reducing the work and costs associated with early oral vaccine development. With this in mind, we take a holistic systems biology approach to compare the transcriptional signatures of peripheral blood mononuclear cells isolated from volunteers, who following two oral priming doses with the oral cholera vaccine Dukoral®, had either strong or no vaccine specific IgA responses. Using this bioinformatical method, we identify *TNFRSF17*, a gene encoding the B cell maturation antigen (BCMA), as a candidate biomarker of oral vaccine induced IgA immune responses. We then assess the ability of BCMA to reflect oral vaccine induced primary and memory IgA responses using an ELISA BCMA assay on a larger number of samples collected in clinical trials with Dukoral® and the oral enterotoxigenic *Escherichia coli* vaccine candidate ETVAX. We find significant correlations between levels of BCMA and vaccine antigen-specific IgA in antibodies in lymphocyte secretion (ALS) specimens, as well as with proportions of circulating plasmablasts detected by flow cytometry. Importantly, our results suggest that levels of BCMA detected early after primary mucosal vaccination may be a biomarker for induction of long-lived vaccine specific memory B cell responses, which are otherwise difficult to measure in clinical vaccine trials. In addition, we find that ALS-BCMA responses in individuals vaccinated with ETVAX plus the adjuvant double mutant heat-labile toxin (dmLT) are significantly higher than in subjects given ETVAX only. We therefore propose that as ALS-BCMA responses may reflect the total vaccine induced IgA responses to oral vaccination, this BCMA ELISA assay could also be used to estimate the total adjuvant effect on vaccine induced-antibody responses, independently of antigen specificity, further supporting the usefulness of the assay.

## Introduction

Enteric disease remains a leading cause of mortality and morbidity in low- and middle-income countries. Whilst most enteric infections could likely be controlled by orally administered mucosal vaccines, only a few oral vaccines are currently licensed for human use. This is because in part, measuring mucosal immune responses to enteric pathogens and mucosal vaccines is more challenging than parentally administered vaccines (1, 2).

Though there is no known absolute correlate of protection, the induction of IgA antibodies in the intestinal mucosa is considered essential for protection against enteric microorganisms (3, 4). One standard measurement of mucosal immune responses in oral vaccine trials is to quantify IgA antibody secreting cell (ASC) responses in peripheral blood mononuclear cells (PBMCs) using ELISPOT or antibody in lymphocyte supernatants (ALS) (3, 5). By assessing vaccine-specific ASCs five days after either the second oral priming or any booster vaccine dose, vaccine-specific mucosal B cell responses can be optimally estimated (6, 7).

However, analyzing gut homing ASC responses to multivalent oral vaccines is practically demanding and labor intensive. Using tools that simplify the isolation of PBMCs and the ALS assay instead of the traditional ELISPOT technique significantly reduces the work-load. But assessing antibodies specific for the different components of multivalent vaccines can still be challenging, because by only focusing on responses to well-defined antigens, the overall immunogenicity or adjuvanticity of the whole cell vaccine may be missed. Consequently, a useful tool to aid oral enteric vaccine development would be to identify a biomarker which reflects overall vaccine immunogenicity (3).

Systems biology, that employs “omics” technologies combined with conventional immunological readouts can assist in the holistic identification of predictive vaccine immune signatures (8, 9). Thus system biology studies have identified genetic signatures predictive of antibody responses to parenteral vaccines against yellow fever, influenza and tuberculosis (9–13). It remains unknown if such approaches can be used to predict immune responses to oral vaccines against enteric diseases.

Using a systems biology approach, the aim of this study was to identify a practical and relatively low cost biomarker assay to assess oral vaccine induced IgA immune responses in humans. By transcriptionally profiling PBMCs from Swedish volunteers receiving the oral cholera Dukoral® vaccine, we identify *TNF Receptor Superfamily member 17* (*TNFRSF17*), a gene encoding the protein B cell maturation antigen (BCMA) (14) as a candidate biomarker. We then quantify BCMA in samples collected from subjects participating in Dukoral® and clinical trials of an oral enterotoxigenic *Escherichia coli* (ETEC) vaccine (15–18) and demonstrate that BCMA levels in ALS specimens reflect antigen-specific ALS IgA responses to cholera and ETEC vaccines.

## Materials and Methods

### Vaccines

Dukoral® (Valneva Sweden AB, Stockholm) contains 1.25 × 10^11^ inactivated *Vibrio cholerae* O1 bacteria and 1 mg of recombinant cholera toxin B subunit (CTB). ETVAX consists of four inactivated recombinant *E. coli* strains that overexpress the ETEC colonization factors CFA/I, CS3, CS5, and CS6, mixed with LCTBA (a hybrid molecule of CTB and heat-labile toxoid B subunit (LTB). ETVAX was administered either alone or with 10 μg of double mutant heat labile toxin adjuvant (dmLT). Both vaccines were administered orally in 150 mL of bicarbonate buffer (16–18).

### Vaccine Immunization Schedules and Immune Assessments

Descriptions and immunological results from the Dukoral® and ETVAX vaccine trials are published elsewhere (16–18). In the Dukoral® vaccine study, healthy Swedish adults were primed with two oral doses of the vaccine two weeks apart (Supplementary Figure 1A); three months later the volunteers received a single booster dose of a full or a reduced dose of Dukoral®.

For O-specific polysaccharide (OSP) ALS IgA antibody analysis, standard binding multi-array plates (Meso Scale Discovery) were coated with 0.3 μg/mL of OSP-BSA Ogawa antigen (19). ALS samples were incubated for two hours on the plates before being analyzed using an electrochemiluminescence assay method (20).

For the ETVAX vaccine studies (Supplementary Figure 1B), healthy Swedish adults were primed with two oral doses, two weeks apart of either vaccine buffer (placebo group) or ETVAX vaccine with or without 10 μg dmLT (16); a subset received an oral ETVAX booster dose after 13–23 months (17). The anti-CFA/I, CS3, CS5, CS6, and LTB. ALS IgA ELISA results from these two ETVAX trials were used in this study for the ALS-BCMA response comparisons (16, 17).

In all the vaccine trials, two-fold rises (FR) in ALS IgA antibody responses were considered as responses (16–18).

### PBMC, ALS and Serum Preparations

Blood samples were collected at the time points indicated in Supplementary Figure 1. PBMCs were isolated by density-gradient centrifugation using Ficoll-Paque and cryopreserved (16–18, 21). In the ALS assay, 2 × 10^6^ fresh PBMCs (200 μL/well) were cultured for 72 hours at 37°C, 5% CO_2_, after which supernatants were collected and stored at −70°C (16– 18).

### RNA Extraction From PBMCs of Dukoral® Vaccinated Volunteers

**Figure 1:**
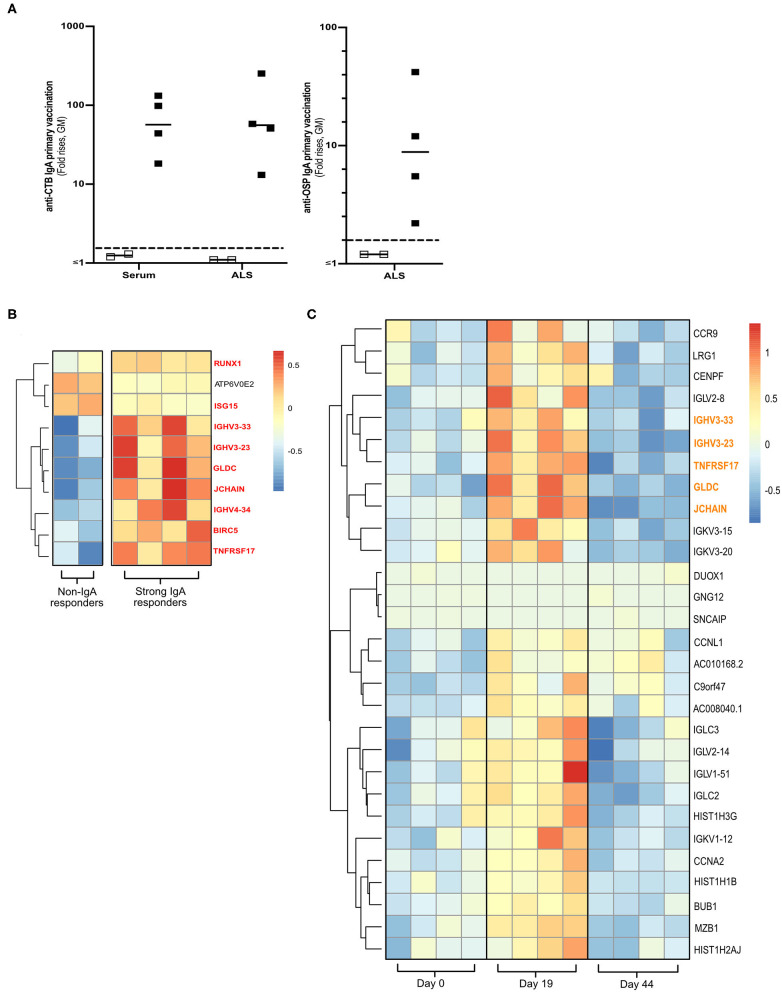
Transcriptomics analysis of PBMCs from Dukoral® vaccinated volunteers. (**A)** RNA-Seq analysis was performed on PBMCs from six Swedish volunteers who following two oral priming doses had either strong (*n* = 4, closed squares) or no (*n* = 2, open squares) IgA responses to the CTB and OSP antigens in the oral cholera vaccine. Volunteers with two-fold increases in IgA antibody responses are considered as vaccine responders (dashed line). Bars represent geometric means. **(B)** Heatmap of DEGs (padj < 0.05) of high vs. non-IgA vaccine responders that were enriched in Reactome pathways at day 19 post-vaccination. Unique DEGs to strong IgA vaccine responders are highlighted in red. **(C)** Heatmap of unique PBMC DEGs (padj < 0.05) that were enriched in Reactome pathway analysis at day 19 post-vaccination. Unique DEGs that were also identified in the strong vs. non-IgA vaccine analysis at day 19 post-vaccination are highlighted in orange. Color scales on Heatmaps; Red = up, White = no change, Blue = down regulated transcripts.

Total RNA was extracted from cryopreserved PBMCs (5 × 10^6^) from four strong and two non-IgA antibody responders of the Dukoral® vaccine study (Figure 1A and Supplementary Figure 1A) (18). The four responders had responded strongly with IgA against both CTB (>18 FR in serum and >13 FR in ALS) and OSP (>2.2 FR in ALS). The two non-IgA responders had anti-CTB IgA FR <1 in ALS and <1.3 in serum, and anti-OSP IgA FR < 1 in ALS following primary vaccination.

PBMCs were thawed rapidly and washed twice. Total RNA was extracted using the RNeasy Mini kit (Qiagen). RNA purity and integrity was assessed using the 2200 TapeStation system Bioanalyser system with RNA ScreenTape and TapeStation Analysis Software (Agilent Technologies, Inc). All RNA samples used for RNA-Seq had a RNA integrity number (RIN) > 9.

### RNA-Seq

RNA-Seq was performed by GeneCore SU, University of Gothenburg, Sahlgrenska Academy, Gothenburg, Sweden (http://www.cgg.gu.se/genecore-su), using the Illumina Nextseq 500 platform (Illumina, San Diego, CA, USA). cDNA libraries were prepared from total RNA samples (≤1,500 ng), using the TruSeq Stranded Total RNA Sample Preparation Kit with Ribo-zero Gold (Illumina Inc.). Each library was paired-end sequenced (2 × 75 bp) using the Illumina Nextseq500 Kit High Output V2.

### Transcriptomics Analysis

All sequencing reads were analyzed for quality control using prinseq/0.20.3 (22). Quality filtered reads were aligned to the Ensembl human reference genome GRCh38.90 using star/2.5.2b (23). In total >49 million paired end sequenced reads of 150 base pairs per sample were obtained, with >83% of these total reads being mapped to the human reference genome (Supplementary Table 1). Read counts mapped toward annotated features were calculated using HTSeq-count/0.6.1p1 (24). Statistical analysis was performed using DESeq2_1.14.1 (25) within R/3.3 software, applying a significance threshold padj < 0.05.

To identify differentially expressed genes (DEGs) in strong vs. non-IgA Dukoral® responders, size factors and dispersion estimates were calculated using the Wald statistical test. To identify unique DEGs of either strong or non-IgA responders, size factors and dispersion estimates were also calculated using the Wald statistical test. Then, a nested design model (25) was used to identify PBMC transcriptional signatures unique to either the strong or the non-IgA vaccine responders five days (i.e., day 19 post-vaccination) after the second oral priming dose. *P*-values for both bioinformatic comparisons were corrected for multiple testing using Benjamini Hochberg. DEGs for both comparisons were saved and used for Reactome pathway enrichment analysis (26). Reactome pathways with a *P* < 0.05 and FDR < 0.05 were considered as significant.

### Flow Cytometric Analysis

Total plasmablasts (defined as CD19^+^CD27^+^CD38^high^ cells) and IgA^+^ plasmablasts were quantified using flow cytometry in cryopreserved PBMCs from a subset of ETVAX vaccinees, including subjects who responded either with strong or weak/no vaccine specific IgA antibodies in ALS specimens, as described (15). At least 500,000 live lymphocytes were analyzed using an LSRII flow cytometer (BD Pharmingen, San Diego, CA) and FlowJo analysis software (Version 10, Treestar Inc., Ashland, OR).

### ALS and Serum BCMA ELISA Analyses

To quantify ALS-BCMA levels, the human BCMA/TNFRSF17 ELISA Duoset kit (R&D, Inc.) was used according to manufacturer's instructions. ALS samples were diluted 1:2, as determined by a pre-linearity dilution test. The mean coefficient of variation (CV) of interpolated values of BCMA in ALS samples was <10% between runs. BCMA in serum was analyzed using a less labor-intensive human BCMA ELISA KIT (ab263875; Abcam); sera were diluted 1:100, as determined by pre-linearity dilution. The Abcam kit demonstrated a consistently low CV (<10%) between runs and gave comparable ALS results to the R&D BCMA ELISA Duoset kit. All samples were tested in duplicates.

### Statistical Analysis

Magnitudes of BCMA and IgA responses in ALS specimens were calculated as post-immunization divided by pre-immunization antibody levels. Subjects with a combined ALS response index >150, defined as the sum of the magnitudes (maximal fold rises above the pre vaccination levels) of ALS IgA responses to the five major vaccine antigens LTB, CFA/I, CS3, CS5, and CS6, were classified as strong vaccine responders whereas subjects with a response index <135 were classified as weak/non-responders.

Data was evaluated with GraphPad Prism version 8.0 (GraphPad Software, Inc). Pre- and post-vaccination ALS-BCMA levels were compared using either the Wilcoxon signed-rank or Mann Whitney U test. The Spearman Rank correlation coefficient test was used to compare magnitudes of ALS BCMA and ALS IgA antibody responses or either total plasmablasts or IgA+ plasmablasts. Associations between combined ALS IgA responder indexes and magnitudes of ALS BCMA responses were evaluated using Fisher's exact test. *P* < 0.05 were considered significant.

## Results

### Transcriptomic Profiling of PBMCs Collected From Participants in an Oral Cholera Dukoral® Vaccine Clinical Trial

To identify a biomarker of oral vaccine IgA responses in humans, cryopreserved PBMCs were selected from a subset of volunteers who following primary Dukoral® vaccination (Supplementary Figure 1A and Figure 1A) had high (*n* = 4) or low (*n* = 2, non-responders) IgA responses to the CTB and OSP antigens (18). Total RNA was extracted and paired end RNA-seq was performed to generate whole PBMC transcriptomic datasets.

Using these transcriptional PBMC datasets, we identified DEGs (multiplicity adjusted *P*-value [padj] < 0.05) in the PBMCs of strong vs. non-IgA vaccine responders at days 0, 19 and 44 post vaccination (Supplementary Table 2), before performing a pathway enrichment analysis using Reactome (26). This Reactome analysis only identified significantly enriched pathways (*P* < 0.05 with false discovery rate [FDR] < 0.05) in the PBMC DEG datasets of high vs. non-IgA vaccine responders at day 19 post Dukoral® vaccination (Supplementary Table 3), where the top ten of these enriched pathways were found to be associated with the general development of B cells and antibody responses. Enriched PBMC DEGs of high vs. non-IgA vaccine responders in these Reactome over-represented pathways (Figure 1B) included: *runt-related transcription factor 1* (*Runx1*), a master transcriptional regulator of hematopoietic stem cells differentiation (27) and *J chain*, encoding a protein essential for polymeric IgM and IgA production and polymeric Ig receptor-mediated epithelial transport of IgA and *TNFRSF17*, a member of the tumor necrosis superfamily (14).

### PBMC Transcriptome Profiling Identifies a Candidate Biomarker Associated With IgA Antibody Responses to Oral Cholera Dukoral® Vaccination

Next, we assessed which PBMC transcriptional signatures were unique to either the strong or the non-IgA vaccine responders five days (i.e., day 19 post-vaccination, Supplementary Figure 1A) after the second oral priming dose, when maximum plasmablast/ASC responses are observed in the circulation (6, 7, 16). By applying a multi-variable nested design statistics model (25) and performing Reactome enrichment analysis, unique day 19 post-vaccination PBMC DEGs were found to be associated with B cell development and antibody responses (Supplementary Table 4). These DEGs (Figure 1C) included the gut lymphocyte homing encoding molecule C–C motif chemokine receptor 9 (*CCR9*) (14) and the previously identified genes in the high vs. non-IgA responder analysis (Figure 1B in red); *J chain, Immunoglobulin heavy variable 3-23* (*IGHV3-23*), *IGHV3-33, Glycine dehydrogenase* (*GDLC*) and *TNFRSF17*.

Notably, *TNFRSF17* encoding BCMA protein, is expressed in activated B cells committed to plasma cell differentiation (14). *TNFRSF17* has also been shown to be a predictor of neutralizing antibody responses to various parental vaccines in other system biology studies (10–12, 28). We therefore decided to focus on BCMA in our continued analyses, aiming to determine if BCMA could be used as a biomarker of oral vaccine-induced IgA immune responses.

### Validation of the BCMA Marker Using ALS Specimens From the Oral Cholera Dukoral® Vaccine Trial

Membrane-bound BCMA (encoded by *TNFRSF17*) is spontaneously cleaved off activated B cells, leading to a detectable soluble form in human blood and supernatants of cell cultured PBMCs (29, 30). We therefore validated our PBMC transcriptional analysis by quantifying BCMA in ALS specimens from the Dukoral® vaccine study using commercially available BCMA ELISA kits.

**Figure 2:**
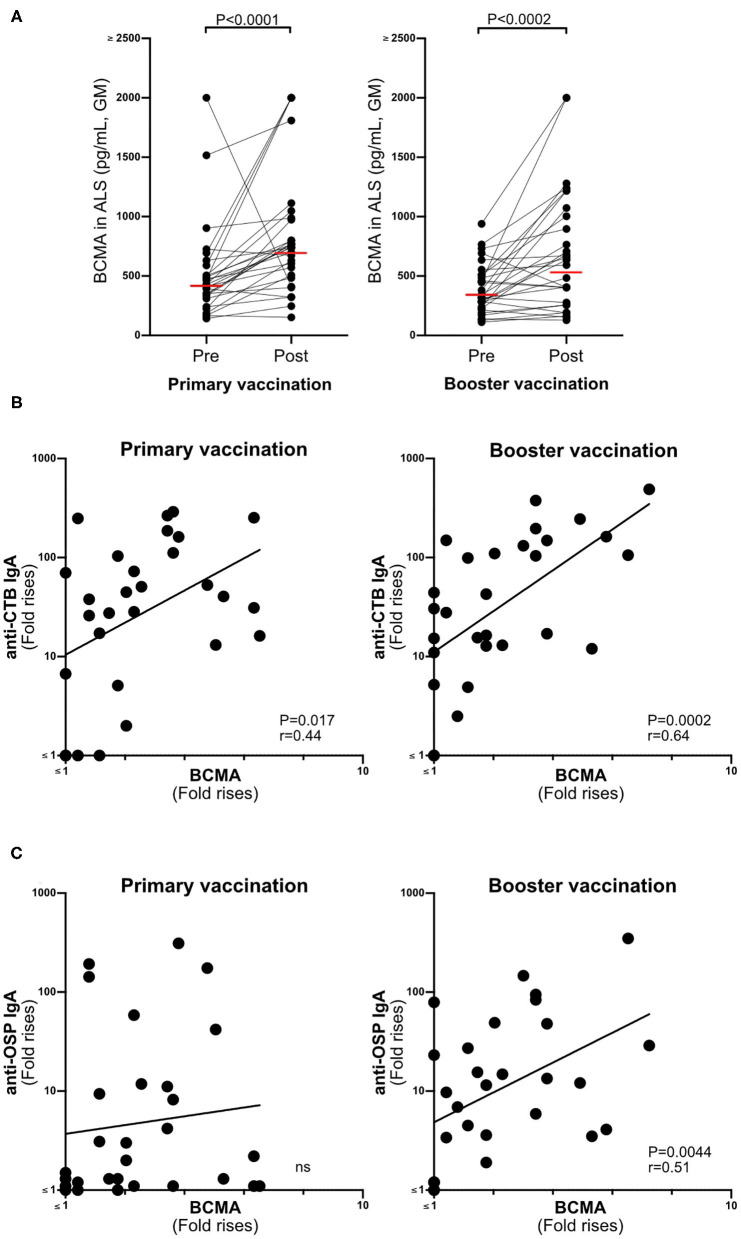
BCMA responses in ALS specimens correlate with Dukoral® ALS vaccine responses in Swedish volunteers. **(A)** Concentrations of BCMA (pg/mL) in ALS specimens were quantified by ELISA in 29 individuals before (pre) and after (post) primary and booster Dukoral® vaccination. Horizonal bars represent geometric mean values. **(B)** Correlation between the magnitudes of ALS CTB IgA responses and ALS BCMA fold rises following primary and booster Dukoral® vaccinations. **(C)** Correlation between the magnitudes of ALS OSP IgA and ALS BCMA responses following primary and booster Dukoral® vaccinations. Each symbol represents one individual.

BCMA concentrations were significantly higher in ALS samples (*n* = 29) collected after primary and booster Dukoral® vaccination compared to prevaccination (Figure 2A). There were also significant correlations between magnitudes of ALS-BCMA responses following primary (i.e., day 19/day 0) and booster (i.e., day 5/day 0) vaccination and with the magnitudes of anti-CTB ALS IgA responses (Figure 2B). Magnitudes of ALS BCMA responses however only correlated with anti-OSP IgA responses following booster vaccination (Figure 2C), where 79% of subjects were responders to OSP. In comparison, only 52% of subjects were responders to OSP following primary vaccination, which may explain the lack of correlation between BCMA and IgA levels after primary vaccination.

### ALS BCMA Is Higher in Oral Vaccinees Than in Placebo Recipients

To verify our findings in another independent oral vaccine trial, we assessed magnitudes of BCMA responses in ALS and serum samples collected from Swedish adults who participated in trials of the ETEC candidate vaccine ETVAX. ALS and serum samples were used from volunteers who were primed with two oral doses of either vaccine buffer alone (placebo group, *n* = 21) or ETVAX administered with or without 10 μg of dmLT adjuvant (*n* = 47, Supplementary Figure 1B) (16).

**Figure 3:**
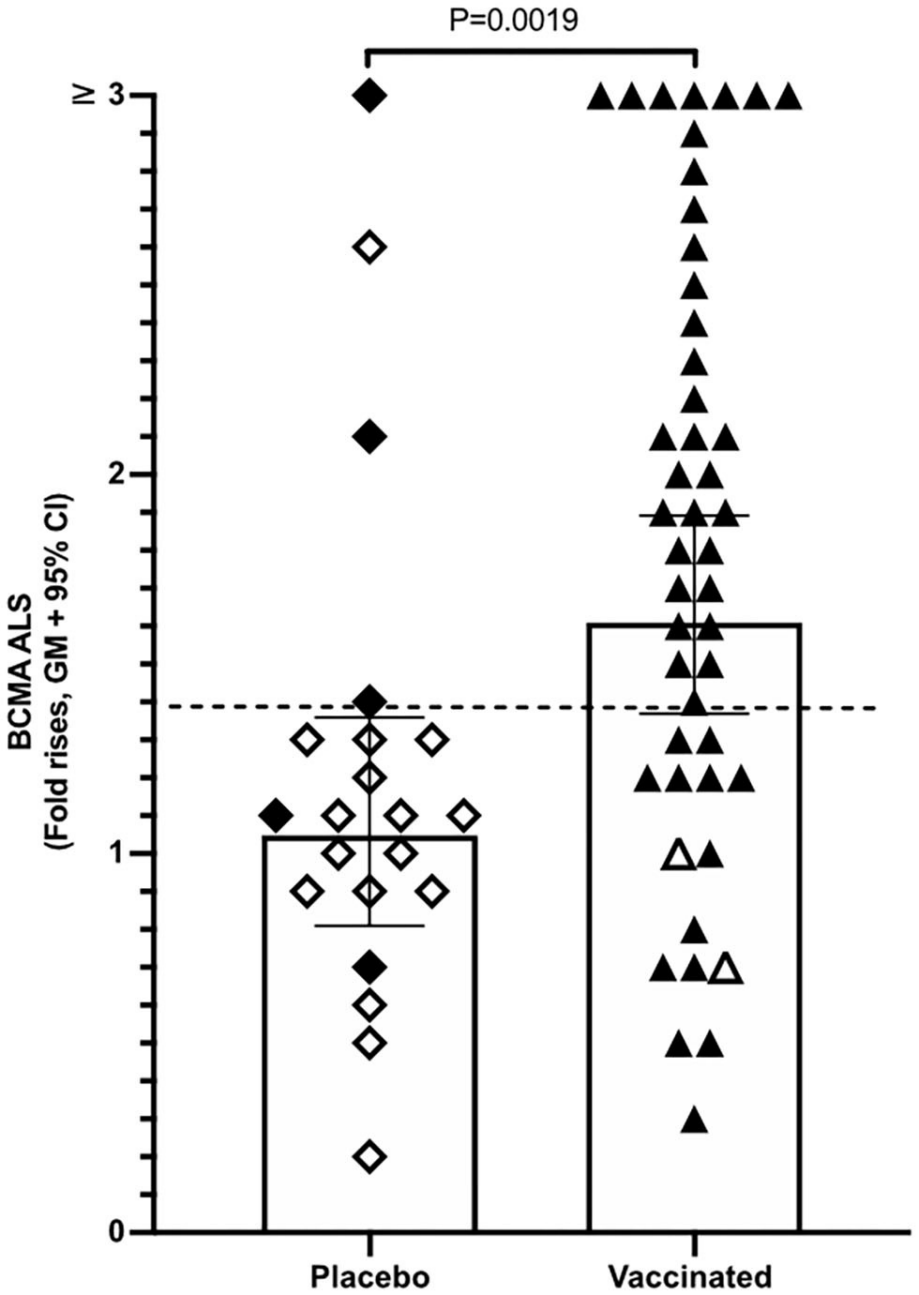
ALS BCMA responses is higher in orally vaccinated subjects than in placebo recipients. Magnitudes of ALS BCMA responses from volunteers were primed with 2 oral doses 2 weeks apart (day 0 and day 14), of either vaccine buffer (placebo group, *n* = 21), or the oral ETVAX vaccine, administered with or without 10 μg dmLT (*n* = 46). Each symbol represents one individual. Filled diamonds or triangles represent individuals who responded to at least one ETVAX vaccine antigen in ALS IgA. Open diamonds and triangles represent subjects that did not respond to any of the vaccine antigens in ALS. ALS BCMA responses higher than the arbitrarily chosen level of 1.4 is indicated by a dashed line. Bars represent geometric means with 95% CI.

Magnitudes of BCMA responses were significantly higher in ALS specimens of ETVAX vaccinated individuals compared to placebo recipients (Figure 3). Moreover, individuals who responded with ALS IgA antibodies to at least one of the ETVAX vaccine antigens (indicated by black diamonds or triangles in Figure 3) more frequently (*P* = 0.0320) had magnitudes of ALS BCMA responses with geometric titres >1.4. In contrast, subjects who did not respond to any of the vaccine antigens (indicated by open diamonds or triangles) more frequently had geometric mean titres <1.3. We found no significant differences between the magnitudes of serum-BCMA responses in placebo and ETVAX vaccinated individuals at any time-point post-vaccination (Supplementary Figure 2).

### ALS BCMA Responses Accurately Reflect and Predict IgA Responses to an Oral ETEC Vaccine Candidate

**Figure 4:**
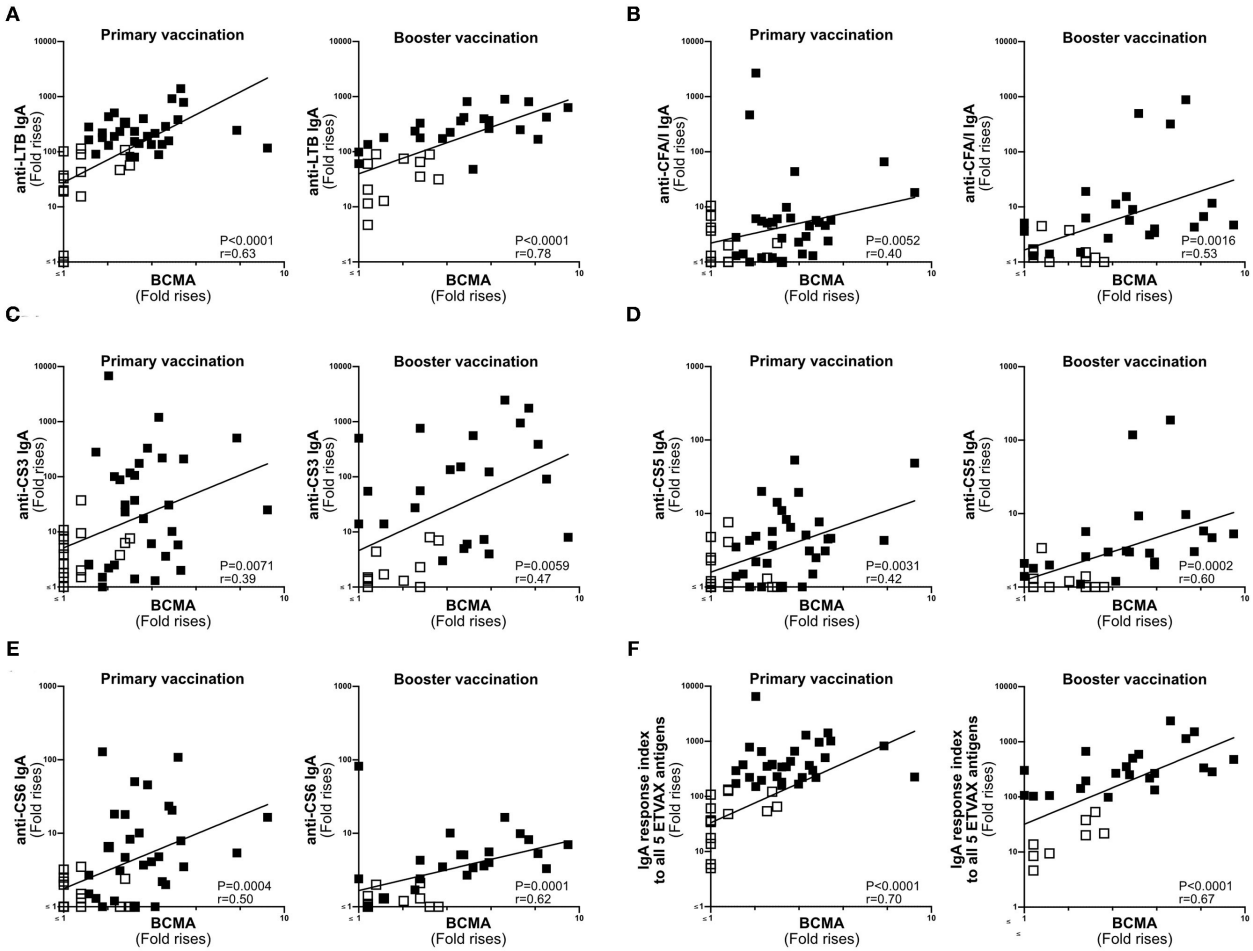
ALS BCMA responses correlate with ALS IgA responses to ETVAX vaccine antigens. Correlations between the vaccine specific ALS IgA responses against each individual ETVAX vaccine antigen **(A–E)** and magnitudes of ALS BCMA responses following primary (*n* = 46) and booster (*n* = 35) ETVAX vaccination. **(A)** LTB IgA, **(B)** CFA/I IgA, **(C)** CS3 IgA, **(D)** CS5 IgA, **(E)** CS6 IgA. **(F)** Correlations between the combined ALS IgA responses against the five major ETVAX vaccine antigens (response index) and the magnitudes of ALS BCMA responses following primary (*n* = 46) and booster (*n* = 35) ETVAX vaccination. Each symbol represents one individual. Filled squares represent vaccine responders with an ALS IgA combined response index >150. Open squares represent vaccine responders with an ALS IgA combined response index <135.

Next, we compared the magnitudes of BCMA and vaccine-specific IgA responses in ALS specimens of individuals primed with two doses of ETVAX with or without 10 μg dmLT (*n* = 47) (16), followed by a single booster dose of ETVAX 13–23 months later (*n* = 35, Supplementary Figure 1B) (17). Magnitudes of ALS-BCMA responses and the maximal magnitude of ALS-IgA responses (measured any day post-vaccination compared to pre-vaccination) correlated significantly against each of the five individual ETVAX antigens (Figures 4A–E). Likewise, magnitudes of ALS-BCMA responses directly correlated (*P* < 0.006) with magnitudes of ALS-IgA responses on day 19 post-vaccination (Supplementary Figure 5).

**Figure 5:**
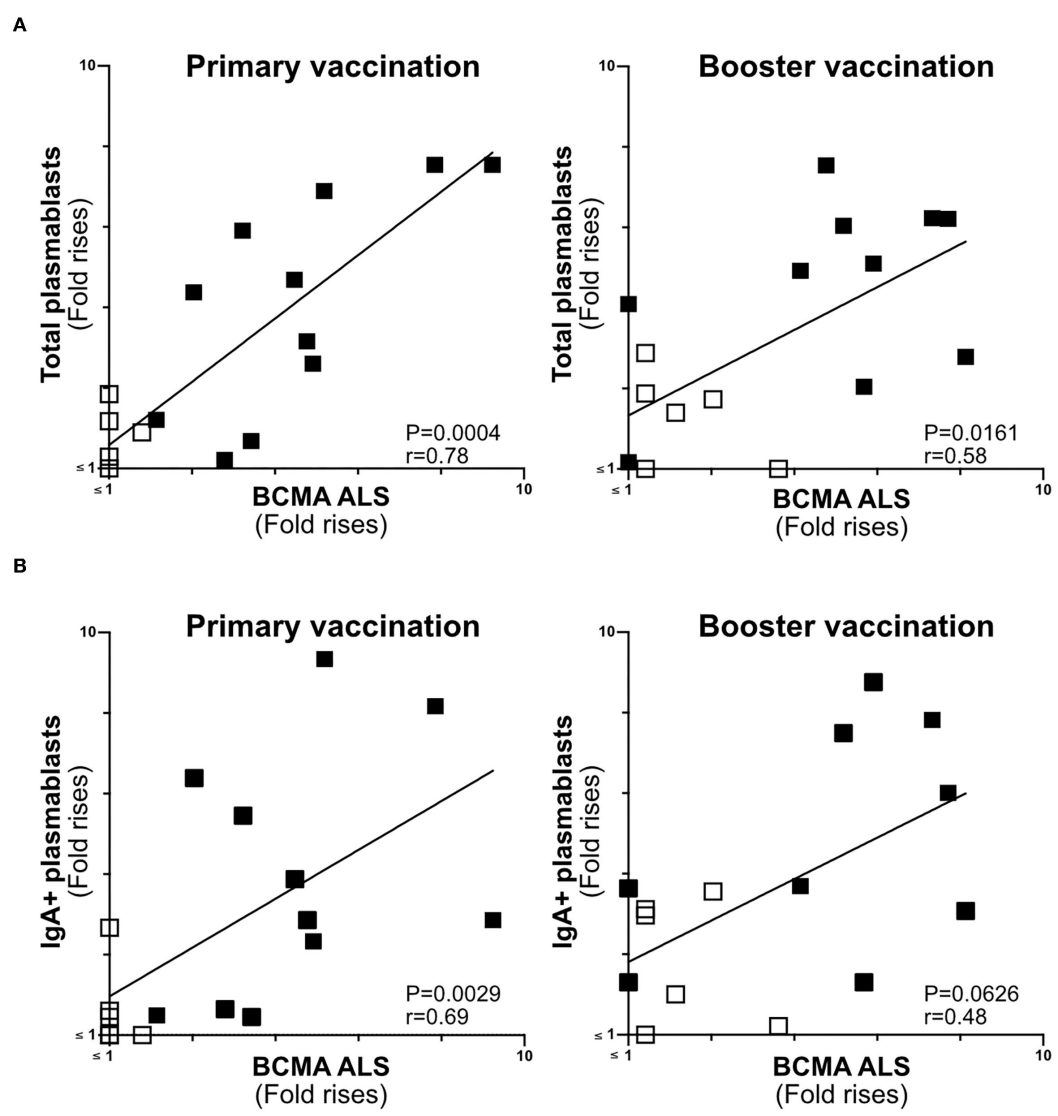
ALS BCMA response magnitudes correlate with ETVAX induced plasmablast responses. **(A)** Correlations of the magnitudes of total plamablast responses (fold rises in frequencies of CD19^+^CD27^+^CD38^high^ cells among cells detected by flow cytometric analysis in post compared to pre-vaccination samples) induced by primary and booster ETVAX vaccination with ALS BCMA responses (*n* = 17). **(B)** Correlations of the magnitudes of IgA^+^ plamablast responses induced by primary and booster ETVAX vaccination with ALS BCMA responses (*n* = 16). Each symbol represents one individual. Filled squares represent vaccine responders with an ALS IgA combined response index >150. Open squares represent vaccine responders with an ALS IgA combined response index <135.

Magnitudes of ALS-BCMA responses also significantly correlated with the combined ALS-IgA responses to all five primary ETVAX vaccine antigens (Figure 4F). We found that a higher proportion (*P* = 0.0001) of strong vaccine responders (combined ETVAX ALS IgA response >150, closed squares in Figure 4), had ALS-BCMA response magnitudes >1.4 compared to weak vaccine responders (combined ETVAX response index <135, open squares in Figure 5) who more frequently had ALS-BCMA response magnitudes <1.3.

### The Magnitudes of Plasmablast and ALS-BCMA Responses Correlate in Oral ETEC Vaccinees

BCMA has previously been shown to be strongly expressed on the surface of plasma cells (14, 30). Therefore, we determined if ALS-BCMA responses correlate with the frequencies of plasmablasts/cells using results from a previous flow cytometric analysis of PBMCs collected from a subset (*n* = 17) of the ETVAX vaccinated subjects (15). When we compared the volunteers ALS-BCMA FR with magnitudes (FR in frequencies) of responses among total plasmablasts, we found significant correlations after primary and booster ETVAX vaccination (Figure 5A). Similarly, the magnitudes of ALS BCMA responses directly correlated with IgA+ plamablast responses (Figure 5B) following primary vaccination.

### Application of ALS-BCMA Responses as an Oral Vaccine Biomarker

**Figure 6:**
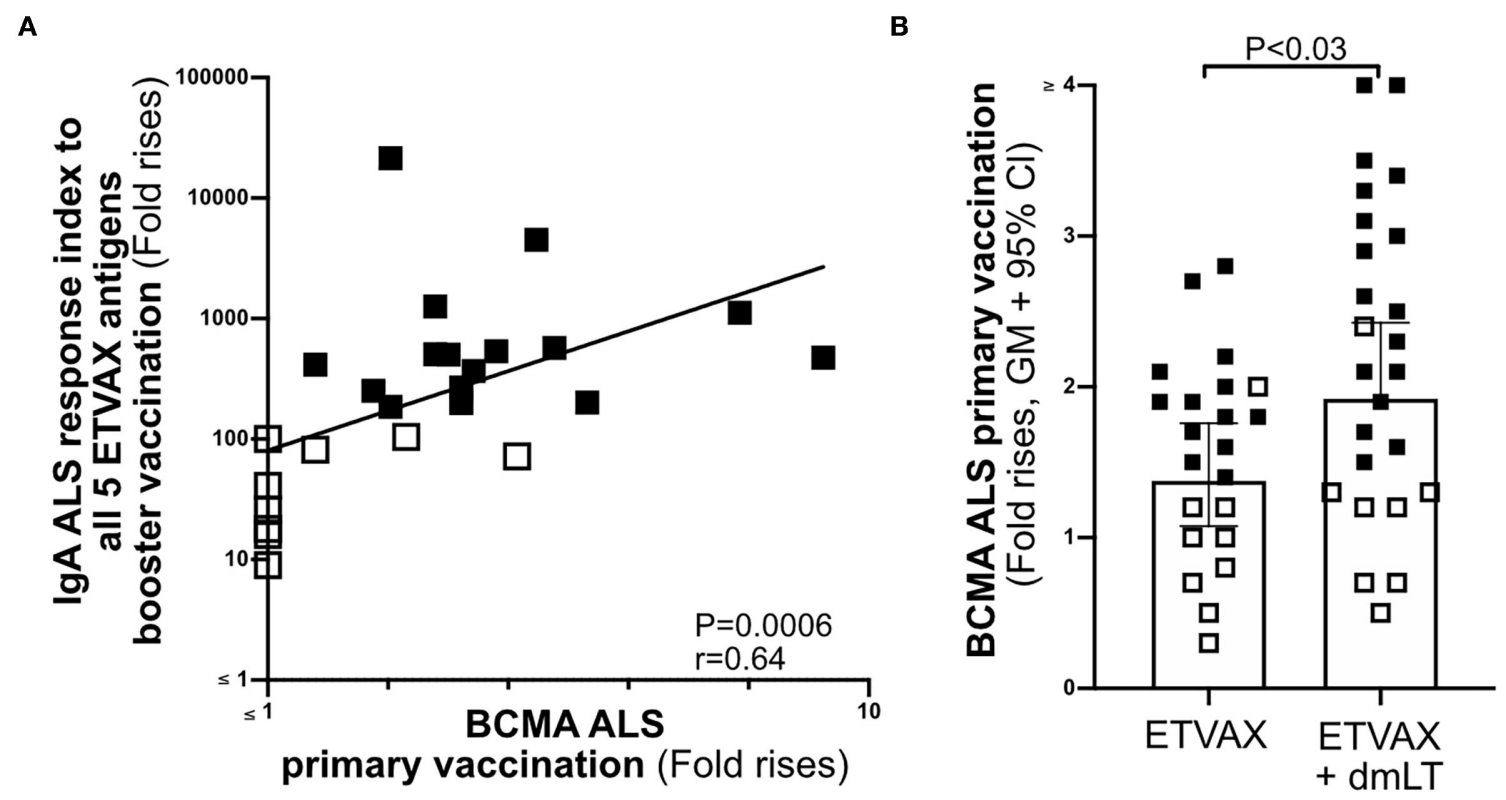
The usefulness of ALS BCMA as an oral vaccination biomarker. **(A)** Correlation between magnitudes of ALS BCMA responses following primary ETVAX vaccination and the combined booster ALS IgA responses to all five major ETVAX vaccine antigens (*n* = 29). **(B)** Magnitudes of ALS BCMA responses in volunteers who received ETVAX with 10 μg dmLT (*n* = 24) compared to ETVAX only (*n* = 21). Bars represent geometric means with 95% CI. Filled squares represent vaccine responders with an ALS IgA combined response index >150. Open squares represent vaccine responders with an ALS IgA combined response index <135.

To establish if the ALS-BCMA biomarker could also be used to predict oral vaccine memory IgA responses, we compared ALS-BCMA responses after primary oral ETVAX vaccination with the ALS-IgA antibody responses of booster vaccinated volunteers 13–23 months later (*n* = 25, Supplementary Figure 1B). We found that magnitudes of ALS-BCMA responses following primary vaccination significantly correlated with ALS-IgA responses to all five ETVAX antigens (combined response index, Figure 6A), and to each individual ETVAX antigen (Supplementary Figure 6) following booster vaccination. We also observed that higher proportions (Table 1, *P* = 0.0001) of strong booster IgA vaccine responders (combined response index >150 after booster vaccination, Figure 6A closed squares) had magnitudes of BCMA responses >1.4 in their ALS samples after primary vaccination, compared to weak vaccine responders (combined response index <135 to booster vaccination, Figure 6A, open squares) who had ALS BCMA responses <1.3.

**Table 1:** Magnitudes of BCMA ALS responses can be used to identify strong and weak IgA vaccine responders.

**Magnitude of ALS BCMA response following primary vaccination**	**Primary vaccination (*****n*** **= 25)**		**Booster vaccination (*****n*** **= 25)**	
	**Non-IgA responders^a^**	**Strong IgA responders^b^**	**Non-IgA responders^a^**	**Strong IgA responders^b^**
	**(*n* = 9)**	**(*n* = 16)**	**(*n* = 8)**	**(*n* = 17)**
<1.3	8	0	7	1
>1.4	1	16	1	16
*P*-value^c^	*P* < 0.0001		*P* < 0.001	

a *ALS IgA combined response index to all five ETVAX vaccine antigens <135*.

b *ALS IgA combined response index to all five ETVAX vaccine antigens >150*.

c *Assessed by Fishers exact test*.

*Fisher's exact test was performed to examine the relationship between ALS BCMA magnitudes >1.4 and <1.3 following primary ETVAX vaccination and the ALS IgA combined response index's >150 and <135 following primary or booster ETVAX vaccination*.

Finally, to investigate if ALS-BCMA responses reflect dmLT adjuvanticity, we compared magnitudes of ALS-BCMA responses in subjects who received two priming doses of ETVAX (*n* = 21) with individuals who received ETVAX plus 10 μg dmLT (*n* = 24). We found that ALS-BCMA fold rises in the ETVAX plus dmLT vaccinated group were significantly higher (Figure 6B) than in the group given ETVAX only.

## Discussion

One way to facilitate enteric disease vaccine development could be identify a simple biomarker assay that reflects the development of strong and long-lived vaccine specific IgA immune responses in humans (3). As immune protection to enteric diseases is likely provided by mucosally derived IgA antibodies (31), we took a holistic systems biology approach (9) to identify *TNFRSF17* (BCMA) as a candidate human biomarker of oral vaccine-induced IgA responses. We confirmed the suitability of BCMA to reflect vaccine induced IgA responses by demonstrating that ALS-BCMA ELISA results correlated with ALS-IgA ELISA and flow cytometric plasmablast results obtained in previous oral cholera and ETEC vaccine trials (15–18). Moreover, we found ALS-BCMA could be an early, and easily assessable biomarker for memory B cell induction, as well be a useful biomarker to reflect the overall oral adjuvanticity of dmLT.

The gene *TNFRSF17* (BCMA) has been identified in other systems biology studies as a predictor of antibody responses to parental vaccination (10–12). BCMA, a member of the TNF receptor superfamily is expressed on activated B cells committed to plasma cell differentiation. It might also aid plasma cell survival (14). BCMA binds to the cytokine ligand BAFF, leading to the activation of MHC class II T cell dependent humoral mechanisms (14, 32). BCMA also likely aids IgA class-switching through T cell independent mechanisms (33).

As the enzyme γ-secretase cleaves BCMA off activated B cells, leading to the formation of soluble BCMA in blood and in the media of cell cultured PBMCs (30), we established if BCMA could be used as a human biomarker assay to reflect the quality of overall vaccine specific IgA responses to one or multiple vaccine antigens. We found ALS-BCMA concentrations were significantly higher following primary and booster oral cholera vaccination than before vaccination, with ALS-BCMA responses directly correlating with magnitudes of CTB ALS-IgA responses. We also found ALS-BCMA responses correlated with ALS-IgA fold rises to all and each individual major antigen component of the oral ETEC vaccine candidate ETVAX. Unlike previously published parenteral vaccine studies (10, 12), we could not detect any differences in serum-BCMA levels between placebo and orally vaccinated Swedish subjects. It is plausible that orally induced vaccine BCMA responses are too weak to be accurately determined in serum. However, it remains to be determined if such serum BCMA measurements could be accurately measured in individuals who live in endemic areas and are naturally primed with cholera and ETEC.

We have previously shown that the strong vaccine specific ALS IgA responses detected on day 4/5 after administration of a single booster ETEC vaccine dose reflects mucosal memory IgA responses (17). In this study, we found that ALS-BCMA fold rises >1.4 were more frequently observed in individuals who were ALS-IgA vaccine responders. In comparison, non-IgA-responders generally had ALS-BCMA fold rises <1.3. Importantly, we also observed that volunteers with ALS-BCMA fold rises >1.4 during primary vaccination also had strong memory IgA responses to booster vaccination. These results suggest that levels of BCMA detected after primary mucosal vaccination may be a useful and easily assessable biomarker for induction of long-lived vaccine specific memory B cells that are often difficult to measure in clinical vaccine trials.

Lastly, we also investigated if the ALS-BCMA biomarker could be used to assess the adjuvant effect of co-administrating vaccine with adjuvant *via* an oral administration route. We show that the magnitudes of ALS-BCMA responses were significantly higher when ETVAX was administered with dmLT compared to when the same vaccine dose was given without dmLT. This is consistent with the adjuvant effect previously observed when dmLT was coadministered with ETVAX both in adults and children and when tested together with in mice (16, 34, 35). Therefore, this ALS-BCMA assay could be used to estimate the total adjuvant effect of dmLT on all antibody responses induced by the vaccine, independently of antigen specificity. The possibility to measure the adjuvanticity of dmLT across different antigen specificities may become useful in several ongoing or planned clinical trials of this adjuvant in combination with different multivalent vaccines (34).

In summary, we took a systems biology approach to identify *TNFRSF17* (BCMA) as a candidate biomarker of oral vaccine induced IgA responses in humans. Then, using a relatively low cost BCMA ELISA assay in combination with the simple ALS culture method, we quantified BCMA in a larger number of clinical samples collected from subjects participating in Dukoral® and ETVAX trials. Our findings demonstrate that ALS-BCMA ELISA analyses can be used to measure oral vaccine-induced primary and memory IgA responses, to one or more vaccine antigens, as well as to assess the total adjuvant effect of dmLT. The use of such a biomarker may complement existing immunogenicity assays and aid the selection and evaluation of promising mucosal vaccine candidates and adjuvants that induce broad and long-lived immune responses both in preclinical studies clinical vaccine trials.

## Data Availability Statement

The datasets presented in this study can be found in online repositories. The names of the repository/repositories and accession number(s) can be found in https://doi.org/10.17044/scilifelab.12213434, 12213434.

## Ethics Statement

All protocols and methods were approved by the Ethical Review Board in Gothenburg Region, and the Swedish Medical Product agency. The studies were performed in accordance with the Declaration of Helsinki and good clinical practice guidelines. The participants provided their written informed consent to participate in these studies. In addition, Dukoral® vaccinated volunteers also gave full written consent for RNA-seq of their PMBCs. The Dukoral® clinical trial is registered in the ISRCTN database as ISRCTN 11806026. The ETVAX clinical trials are registered as ISRCTN913630076 and ISRCTN27096290. Written informed consent was obtained from the individual(s) for the publication of any potentially identifiable images or data included in this article.

## Author Contributions

LM initiated and planned the study, performed the experiments and analyzed the data. A-MS, AL, and SL provided data and samples from previous vaccine trials. LM and SL wrote the manuscript. All authors contributed to study design, interpretation of data and reviewed the manuscript before submission.

## Conflict of Interest

The authors declare that the research was conducted in the absence of any commercial or financial relationships that could be construed as a potential conflict of interest.
