# Supplementary material for: A Systems Biology Approach Identifies B Cell Maturation Antigen (BCMA) as a Biomarker Reflecting Oral Vaccine Induced IgA Antibody Responses in Humans
Source: Front Immunol. 2021 Mar 22;12:647873. doi: 10.3389/fimmu.2021.647873 (PMC8019727; doi:10.3389/fimmu.2021.647873)
Supplement: Supplementary file 1 [file Data_Sheet_1.docx]

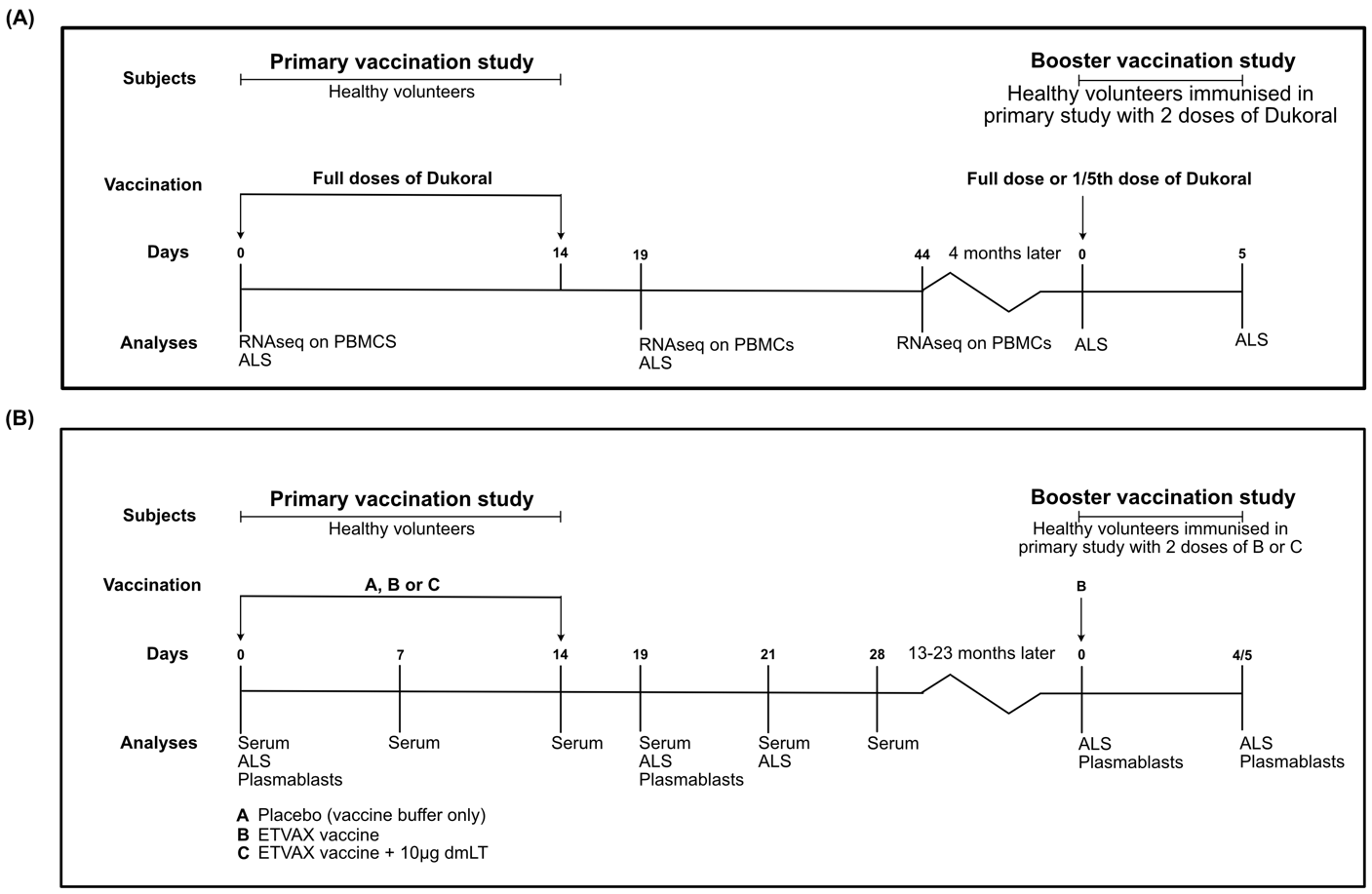


**Supplementary Figure 1. Dukoral^®^ and ETVAX study designs.** (A) In the Dukoral^®^ primary vaccination study, subjects were primed with two oral doses of Dukoral^®^ 14 days apart. Four months later, subjects received a single booster dose of Dukoral^®^. Cryopreserved PBMCs isolated on days 0,19 and 44 after primary vaccinations from 6 volunteers were used for RNAseq analysis. ALS specimens from volunteers collected at days 0, 19 and 44 after primary vaccination and 0 and 5 days after Dukoral^®^ vaccination were used for BCMA and IgA ELISA analysis. (B) For the ETVAX primary vaccination study, subjects were primed with two oral doses two weeks apart of either vaccine buffer (placebo group), or ETVAX vaccine with/without 10µg dmLT. 13-23 months later subjects received a single booster dose of ETVAX. ALS and serum specimens collected at days 0, 7, 14, 19, 21 and 28 after ETVAX vaccination and days 0, 4 and 5 after booster vaccination were used for BCMA and immunological assessments. Flow cytometric analysis of plasmablasts were performed on days 0, 7, 19 and 21 after primary ETVAX vaccination and on days 0,4/5 and 7 after booster ETVAX vaccination.

**Supplementary Figure S2.** **BCMA concentrations in serum specimens from ETVAX vaccinated volunteers.** Concentrations of serum BCMA (pg/mL) was quantified by ELISA in serum specimens collected from volunteers receiving either vaccine buffer alone (placebo group), or ETVAX vaccine with or without 10 μg dmLT on days 0, 7, 14, 19, 21 and 28 post vaccination. Symbols represent geometric mean values for each vaccination group with 95% CI (n=10 per group)

**
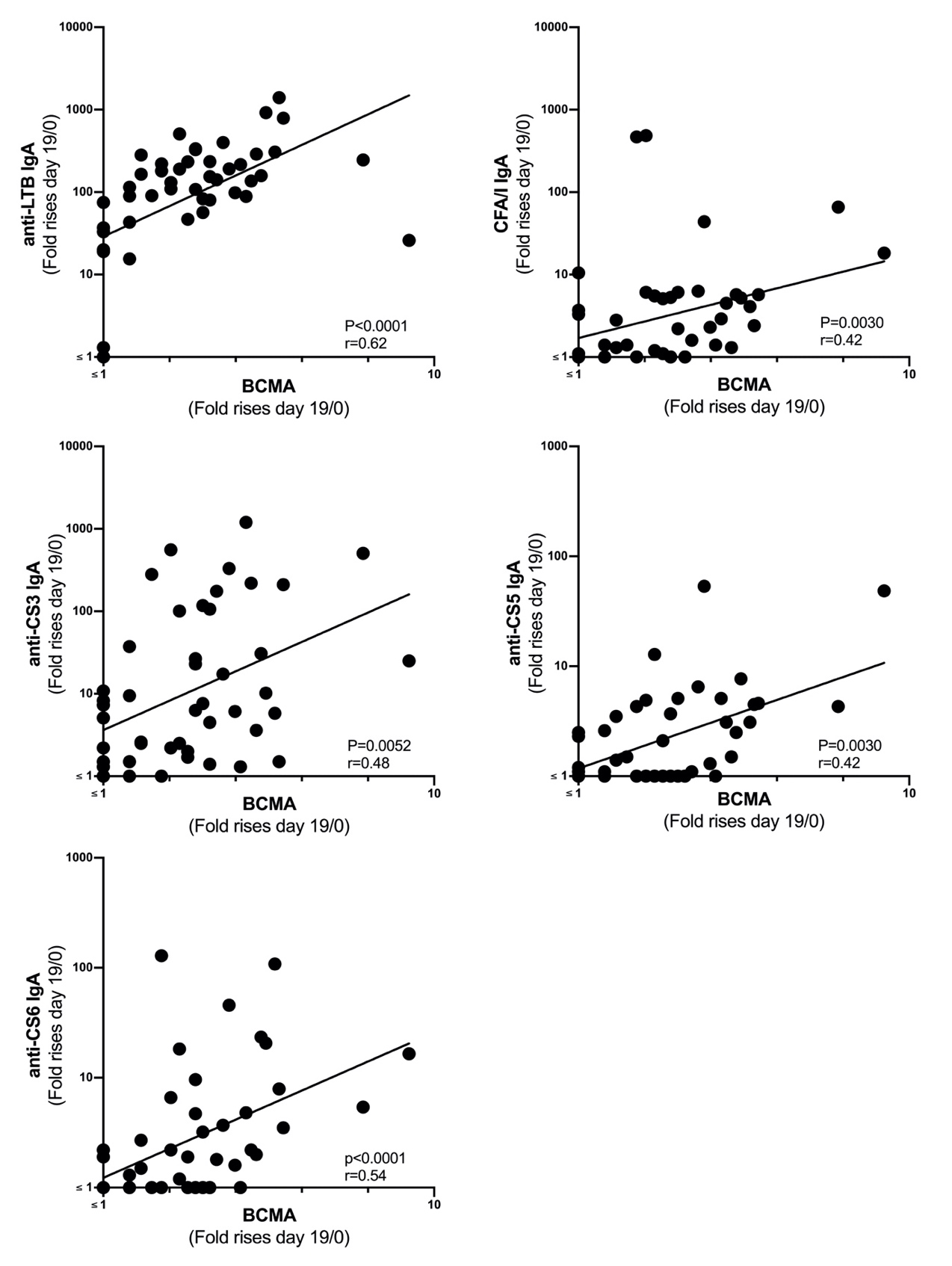
**

**Supplementary Figure S3. ALS BCMA response magnitudes correlate with magnitudes of ALS IgA responses to individual ETVAX vaccine antigens.** Correlations between the magnitudes of vaccine specific ALS IgA responses against each ETVAX vaccine antigen and magnitudes of ALS BCMA following primary (day 19/day 0, n=46) ETVAX vaccinations. Each symbol represents one individual.

**
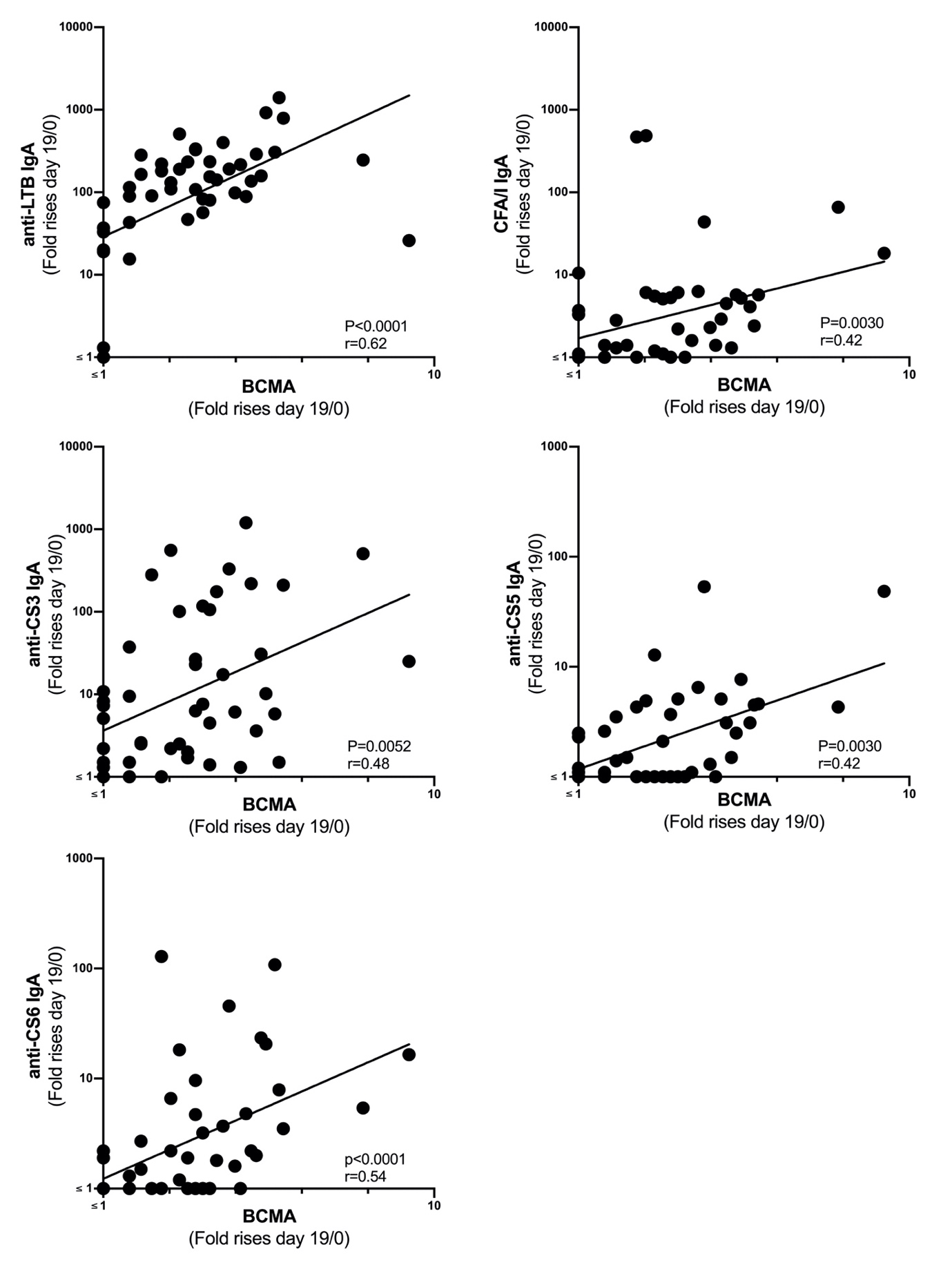
Supplementary Figure S4.** a) to e) ALS BCMA response magnitudes following primary ETVAX vaccination correlates with magnitudes of booster ALS IgA responses to each individual ETVAX vaccine antigen (n=29).

| **Supplementary Table S1** | |  |  |
| --- | --- | --- | --- |
|  |  |  |  |
| Numer of sequenced reads mapped to Enembl human reference genome GRCh38.90 | | | |
|  |  |  |  |
| **Sample name** | **Total number of reads mapped (millions)** | **% aligned** | **Number of aligned uniquely mapped reads (millions)** |
| p1 day 0 | 54.1 | 85.5 | 21.0 |
| p1 day 19 | 51.5 | 86.1 | 20.6 |
| p1 day 44 | 52.5 | 85.4 | 20.7 |
| p2 day 0 | 54.0 | 84.3 | 20.4 |
| p2 day 19 | 48.8 | 85.3 | 19.4 |
| p2 day 44 | 51.0 | 86.5 | 20.5 |
| p3 day 0 | 53.9 | 85.3 | 20.8 |
| p3 day 19 | 50.0 | 85.9 | 19.8 |
| p3 day 44 | 53.6 | 84.1 | 20.4 |
| p4 day 0 | 51.9 | 84.7 | 19.7 |
| p4 day 19 | 53.0 | 84.0 | 20.7 |
| p4 day 44 | 52.6 | 85.2 | 21.1 |
| p5 day 0 | 54.5 | 85.5 | 21.2 |
| p5 day 19 | 51.2 | 84.1 | 19.4 |
| p5 day 44 | 52.6 | 83.0 | 20.2 |
| p6 day 0 | 62.2 | 85.2 | 22.8 |
| p6 day 19 | 54.2 | 85.1 | 20.8 |
| p6 day 44 | 50.9 | 82.1 | 18.6 |
|  |  |  |  |

**Supplementary Table** **S2**

**Supplementary Table S3**

Top 10 enriched Reactome pathways identified in the DEGs (padj <0.05) of strong IgA responder’s versus non-IgA responders at day 19 post Dukoral^®^ vaccination.

| **Identity of enriched Reactome pathway** | ***p*-value** | **FDR^a^** |
| --- | --- | --- |
| R-HSA-895191; RUNX3 regulates RUNX1-mediated transcription | 1,65E-05 | 9,78E-04 |
| R-HSA-2168880; Scavenging of heme from plasma | 1,66E-05 | 9,78E-04 |
| R-HSA-2173782; Binding and Uptake of Ligands by Scavenger Receptors | 9,63E-05 | 0,004 |
| R-HSA-5690714; CD22 mediated B Cell Receptor (BCR) regulation | 1,60E-04 | 0,005 |
| R-HSA-173623; Classical antibody-mediated complement activation | 3,83E-04 | 0,006 |
| R-HSA-2029481; Fc gamma receptor (FCGR) activation | 4,56E-04 | 0,006 |
| R-HSA-983695; Antigen activates B Cell Receptor (BCR) leading to generation of second messengers | 4,56E-04 | 0,006 |
| R-HSA-202733; Cell surface interactions at the vascular wall | 4,95E-04 | 0,006 |
| R-HSA-2730905; Role of LAT2/NTAL/LAB on calcium mobilization | 5,09E-04 | 0,006 |
| R-HSA-166786; Creation of C4 and C2 activators | 5,66E-04 | 0,006 |

**Supplementary Table S4**

Top 10 enriched Reactome pathways identified in the unique DEGs (padj <0.05) of strong IgA responders at day 19 post Dukoral^®^ vaccination.

| **Enriched Reactome pathway identity** | ***p*-value** | **FDR^a^** | |
| --- | --- | --- | --- |
| R-HSA-5690714; CD22 mediated B Cell Receptor (BCR) regulation | 1,09E-12 | | 2,08E-10 |
| R-HSA-2168880; Scavenging of heme from plasma | 1,67E-12 | | 2,08E-10 |
| R-HSA-173623; Classical antibody-mediated complement activation | 1,97E-11 | | 1,63E-09 |
| R-HSA-2029481; Fc gamma receptor (FCGR) activation | 3,51E-11 | | 1,76E-09 |
| R-HSA-983695; Antigen activates B Cell Receptor (BCR) leading to generation of second messengers | 3,51E-11 | | 1,76E-09 |
| R-HSA-2730905; Role of LAT2/NTAL/LAB on calcium mobilization | 5,07E-11 | | 2,08E-09 |
| R-HSA-166786; Creation of C4 and C2 activators | 7,23E-11 | | 2,53E-09 |
| R-HSA-166663; Initial triggering of complement | 1,53E-10 | | 4,74E-09 |
| R-HSA-2173782; Binding and Uptake of Ligands by Scavenger Receptors | 2,02E-10 | | 5,23E-09 |
| R-HSA-2871796; FCERI mediated MAPK activation | 2,09E-10 | | 5,23E-09 |

| **Supplementary Table S5** | |  |  |
| --- | --- | --- | --- |
|  |  |  |  |
| Unique genes in Strong IgA responders at day 19 post vaccination (padj <0.05) | | |  |
|  |  |  |  |
| **Ensembl ID** | **gene name** | **Description** | **Reactome analysis** |
| ENSG00000219410 | AC125494.1 | NA | not found |
| ENSG00000171236 | LRG1 | Leucine rich alpha-2-glycoprotein 1 | found |
| ENSG00000170476 | MZB1 | Marginal zone B and B1 cell specific protein | found |
| ENSG00000148773 | MKI67 | Marker of proliferation Ki-67 | not found |
| ENSG00000211949 | IGHV3-23 | Immunoglobulin heavy variable 3-23 | found |
| ENSG00000178445 | GLDC | glycine decarboxylase | found |
| ENSG00000137857 | DUOX1 | Dual oxidase 1 | found |
| ENSG00000172380 | GNG12 | G protein subunit gamma 12 | found |
| ENSG00000244437 | IGKV3-15 | Immunoglobulin kappa variable 3-15 | found |
| ENSG00000231863 | AL139393.1 | NA | not found |
| ENSG00000211644 | IGLV1-51 | Immunoglobulin lambda variable 1-51 | found |
| ENSG00000048462 | TNFRSF17 | TNF receptor superfamily member 17 | found |
| ENSG00000262874 | C19orf84 | Chromosome 19 open reading frame 84 | not found |
| ENSG00000117724 | CENPF | Centromere protein F | found |
| ENSG00000211679 | IGLC3 | Immunoglobulin lambda constant 3 | found |
| ENSG00000243290 | IGKV1-12 | Immunoglobulin kappa variable 1-12 | found |
| ENSG00000211677 | IGLC2 | Immunoglobulin lambda constant 2 | found |
| ENSG00000222042 | AP001341.1 | NA | not found |
| ENSG00000278196 | IGLV2-8 | Immunoglobulin lambda variable 2-8 | found |
| ENSG00000213694 | C9orf47 | S1P3. Sphingosine-1-phosphate receptor 3 | found |
| ENSG00000263272 | AC004148.1 | Novel transcript, antisense to RPAIN | not found |
| ENSG00000211666 | IGLV2-14 | Immunoglobulin lambda variable 2-14 | found |
| ENSG00000239951 | IGKV3-20 | Immunoglobulin kappa variable 3-20 | found |
| ENSG00000066279 | ASPM | Abnormal spindle microtubule assembly | not found |
| ENSG00000257179 | HMGN2P6 | High mobility group nucleosomal binding domain 2 pseudogene 6 | not found |
| ENSG00000224976 | PARP4P2 | Poly(ADP-ribose) polymerase family member 4 pseudogene 2 | not found |
| ENSG00000226235 | LEMD1-AS1 | LEMD1 antisense RNA 1 | not found |
| ENSG00000276368 | HIST1H2AJ | Histone cluster 1 H2A family member j | found |
| ENSG00000230262 | MIRLET7DHG | Long intergenic non-protein coding RNA 2603 | not found |
| ENSG00000163660 | CCNL1 | Cyclin L1 | found |
| ENSG00000211955 | IGHV3-33 | Immunoglobulin heavy variable 3-33 | found |
| ENSG00000239219 | AC008040.1 | Novel transcript, antisense to SEC62 | found |
| ENSG00000228848 | AC105402.2 | Ribosomal protein S20 (RPS20) pseudogene | not found |
| ENSG00000064692 | SNCAIP | Synuclein alpha interacting protein | found |
| ENSG00000260172 | LINC01413 | Long intergenic non-protein coding RNA 1413 | not found |
| ENSG00000270210 | AC104695.3 | NA | not found |
| ENSG00000279394 | AC015871.5 | NA | not found |
| ENSG00000251636 | LINC01218 | Long intergenic non-protein coding RNA 1218 | not found |
| ENSG00000132465 | JCHAIN | joining chain of multimeric IgA and IgM | found |
| ENSG00000145386 | CCNA2 | Cyclin A2 | found |
| ENSG00000261324 | AC010168.2 | Novel transcript, overlapping HIST4H4 | found |
| ENSG00000214297 | ALDOAP2 | ALDOA pseudogene 2 | not found |
| ENSG00000184357 | HIST1H1B | Histone cluster 1 H1 family member b | found |
| ENSG00000111291 | GPRC5D | G protein-coupled receptor class C group 5 member D | not found |
| ENSG00000279957 | AC110769.3 | TEC | not found |
| ENSG00000173585 | CCR9 | C-C motif chemokine receptor 9 | found |
| ENSG00000258869 | LINC02312 | Long intergenic non-protein coding RNA 2312 | not found |
| ENSG00000240097 | AC104164.2 | Mitochondrial transcription termination factor (MTERF) pseudogene | not found |
| ENSG00000169679 | BUB1 | BUB1 mitotic checkpoint serine/threonine kinase | found |
| ENSG00000251562 | MALAT1 | Metastasis associated lung adenocarcinoma transcript 1 | not found |
| ENSG00000213523 | SRA1 | Steroid receptor RNA activator 1 | not found |
| ENSG00000272033 | AL136984.1 | Novel transcript, sense intronic to POU2F1 | not found |
| ENSG00000227383 | AL353662.1 | Ribosomal protein L35A (RPL35A) pseudogene | not found |
| ENSG00000226292 | AL606495.1 | Ribosomal protein S3A (RPS3A) pseudogene | not found |
| ENSG00000273983 | HIST1H3G | Histone cluster 1 H3 family member g | found |
| ENSG00000167680 | SEMA6B | Semaphorin 6B | not found |
|  |  |  |  |
|  |  |  |  |
| Unique genes in Weak IgA responders at day 19 post vaccination (padj <0.05) | | |  |
|  |  |  |  |
| **Ensembl ID** | **gene name** | **Description** | **Reactome analysis** |
| ENSG00000267724 | AC012254.3 | Novel transcript, antisense to HDHD2 | not found |
| ENSG00000095574 | IKZF5 | IKAROS family zinc finger 5 | not found |
